# Combined transcriptome, metabolome, and miRNA analysis reveals the genetic regulatory network of sweet corn pericarp thickness

**DOI:** 10.3389/fpls.2025.1698281

**Published:** 2025-12-12

**Authors:** Xiaming Wu, Ruixiang Liu, Meijing Zhang, Min Yang, Chenping Zhou, Ruibin Kuang, Yanping Chen, Yuerong Wei

**Affiliations:** 1 Institute of Fruit Tree Research, Guangdong Academy of Agricultural Sciences, Key Laboratory of South Subtropical Fruit Biology and Genetic Resource Utilization, Ministry of Agriculture and Rural Affairs, Guangdong Provincial Key Laboratory of Science and Technology Research on Fruit Tree, Guangzhou, China; 2 Institute of Food Crops, Jiangsu Academy of Agricultural Sciences, Nanjing, China

**Keywords:** metabolism, miRNA, pericarp thickness, sweet corn, transcriptome

## Abstract

The thickness of the pericarp is a complex characteristic that determines sweet corn’s palatability. However, the molecular mechanisms in forming pericarp thickness differences have not been clarified. In this study, we conducted transcriptomics, miRNA analysis, and metabolomics to explore the underlying molecular mechanisms. Scanning electron microscopy (SEM) revealed that the disparity in pericarp thickness is primarily due to variations in the number of cell layers. Our combined multi-omics analysis discovered 6,054 differentially expressed genes (DEGs), 73 differentially expressed miRNAs, 113 differentially accumulated metabolites (DAMs), and several key miRNAs, such as *zma-miR164*, *zma-miR166*, *zma-miR827*, and *zma-miR171b* were identified, which modulate the expression of different transcription factors and regulate the signal transduction of various plant hormones, thereby influencing pericarp thickness. Additionally, our integrated transcriptomic and metabolomic analysis revealed that genes and metabolites involved in plant hormone signal transduction and phenylpropanoids biosynthesis pathway play a significant role in regulating pericarp growth and development. Furthermore, we observed that in the thick pericarp line (M08), the content of cytokinins was significantly reduced, while phenylpropanoid compounds such as 5-O-feruloylquinic acid glucoside, berberine, scopoletin, sinapic alcohol, sinapic acid and 3-O-feruloylquinic acid glucoside accumulated considerably. These findings provide valuable theoretical support and genetic resources.

## Introduction

Sweet corn is a variety of specialized corn that results from one or more genetic mutations in the common maize kernels’ starch synthesis metabolic pathway. These mutations inhibit starch synthesis and lead to the accumulation of sugars such as sucrose (Wu et al., 2020). Sweet corn is high in nutrients, delectable, and has significant economic benefits, making it highly favored by consumers (Swapna et al., 2020).

The sweet corn pericarp consists of three layers: exocarp (outermost, single cell layer), mesocarp (middle, multiple parenchyma cell layers), and endocarp (innermost, closely attached to the seed coat). Its main components include cellulose (35%-45%), hemicellulose (25%-30%), lignin (5%-10%), and pectin (10%-15) (Keegstra, 2010), serving to protect the embryo from physical and biological harm, as well as housing and storing the necessary nutrients for the seedling’s growth (García-Lara et al., 2004). It plays a crucial role in safeguarding the seed’s activity and vitality and is closely linked to the commercial, nutritional, and edible quality of sweet corn. A pericarp that is too thick can impact the corn’s taste, while one that is too thin may lead to ruptures and susceptibility to pathogens (Doll et al., 2017). In China, through the efforts of scientific researchers and breeders, it has developed from the original single low-yielding varieties to high-yielding and high-resistance varieties, with significant achievements in breeding. However, there are still serious shortcomings in quality, especially in thin pericarp varieties (Xiong et al., 2022). Therefore, studying of the pericarp genetic differences can offer valuable guidance for breeding practices.

To date, there has been relatively limited research on the thickness of sweet corn pericarp, with a primary focus on QTL (Quantitative Trait Locus) mapping and genetic analysis. The inheritance of sweet corn pericarp thickness involves dominant effects, additive effects, and epistatic effects, with additive and epistatic effects playing a significant role (Ito and Brewbaker, 1991; Wu et al., 2020). Tracy and Schmidt (1987) discovered that pericarp thickness exhibited a negative correlation with row number, while showing a positive correlation with seed width, cross-sectional size, seed weight, and volume. Previous studies have indicated that QTLs related to sweet corn pericarp thickness have been mapped to all the 10 chromosomes of sweet corn, but most QTLs are challenging to consistently detect in different studies. The South China Agricultural University have mapped QTLs related to sweet corn pericarp thickness to all 10 chromosomes, with major QTLs such as *qPT10-5* (accounting for 7.78%-35.38% of phenotypic variation) and *qPT2–1* identified (Wu et al., 2020; Gong et al., 2024a). Candidate genes for these QTLs include those involved in cell wall synthesis and hormone signaling, but their functional validation remains lacking. Despite the identification of several QTLs related to sweet corn pericarp thickness in previous studies, there has been no report on the cloning of relevant genes to date. Pericarp thickness is influenced not only by genotype but also by environmental factors, kernel traits, and other variables (Khalil and Kramer, 1971; Choe and Rocheford, 2012), making it a complex quantitative trait. Therefore, it is necessary to analyze the genetic regulatory network of pericarp thickness using new methods.

The advancement of high-throughput sequencing has offered a robust technical tool for the extensively identifying of genes associated with specific traits (Gong et al., 2024b; Wu et al., 2024). MicroRNAs are a category of non-coding, single-stranded RNA, typically 21–23 nucleotides in length, which can control the activity of target genes by selectively binding and cleaving mRNA, as well as repressing mRNA transcription. For instance, *miR167* primarily targets auxin response factors (ARFs) involved in plant growth and development (Zhao et al., 2023; Liu et al., 2021). MiR164 regulates NAC transcription factors to affect seed development (Song et al., 2022), and miR166 modulates HD-ZIP genes involved in cell layer formation (Liu et al., 2020), suggesting their potential roles in pericarp thickness regulation. Therefore, in this research, we conducted transcriptome, metabolism, and small RNA sequencing to analyze the differential genes, miRNAs, and metabolites 19 days after pollination of two inbred lines with notable discrepancies in pericarp thickness. Additionally, no studies have integrated transcriptome, miRNA, and metabolome data to systematically explore the regulatory network of pericarp thickness. This is aimed at unveiling the regulatory networks and molecular mechanisms implicated in the formation of pericarp thickness differences, providing a theoretical foundation for the enhancement of sweet corn quality and its industrialization.

## Materials and methods

### Plant material

In the pre-study period, the thickness of the pericarp for 165 sweet corn inbred lines at the milk stage was measured utilizing the micrometer method. Subsequently, several materials with varying pericarp thickness were examined using an electron microscope to validate the accuracy of the data. Eventually, M03 (with a thinner pericarp) and M08 (with a thicker pericarp) were chosen as experimental materials. Each inbred line was planted with 100 plants, with the pistils covered to ensure self-pollination. Sampling was conducted on the 13^th^, 15^th^, 17^th^, 19^th^, 21^th^, 23^th^, 25^th^, and 27^th^ day after pollination, pericarp thickness was measured with 5 biological replicates per stage, each replicate consisting of 5 kernels. A sharp blade was used to cut off the top and base, leaving the middle part of the kernel with a width of 3 mm, followed by a longitudinal cut on the embryonic surface with a blade, so that the pericarp was scored on the embryonic surface, and the pericarp was peeled off with forceps. The pericarp of the dorsal part of the embryos was measured using a micrometer (Mitutoyo 193-101) and frozen for further analysis. For transcriptomic, miRNA-seq, and metabolomic analyses, pericarp samples were collected at 19 DAP, as this stage showed the most significant difference in pericarp thickness between M03 and M08. M03 reaches its maximum pericarp thickness at 15 DAP, but 19 DAP was selected to focus on the stable thickness difference period. Additionally, three biological replicates were used for each analysis, with each replicate consisting of pericarp from 5 kernels of a single ear.

### Scanning electron microscopy phenotype identification

Samples were preserved in centrifuge tubes containing 4% glutaraldehyde. The glutaraldehyde was transferred from the centrifuge tubes to a collection bottle, and then 2% phosphate buffer solution was added, followed by agitation for 10 minutes. This process was repeated three times. Next, 1% osmium tetroxide was introduced to the centrifuge tube and allowed to react for 1.5 hours. Once the reaction was complete, the osmium tetroxide was dispensed into a waste liquid container, and the centrifuge tubes were rinsed with 2% phosphate buffer solution for 10 minutes, a step that was also repeated three times. Following fixation with osmium tetroxide, dehydration was performed by sequentially adding 30%, 50%, 70%, 80%, and 90% ethanol to the centrifuge tubes, with each dehydration step lasting for 10 minutes. Finally, anhydrous ethanol was applied for dehydration for 10 minutes, repeating this process twice to ensure thorough dehydration of the samples in the centrifuge tubes. Place the dehydrated samples in the HCP-2 type critical point dryer for drying 4 hours, then the dried samples were fixed to a clean sample stage using double-sided adhesive. After gold sputtering, the samples were observed using a Hitachi SU8010 field-emission scanning electron microscope (Hitachi High-Technologies Corporation, Tokyo, Japan). Morphological images were captured at multiple magnifications and analyzed using ImageJ software (Version 1.8.0, National Institutes of Health, Bethesda, MD, USA) for quantitative characterization of surface roughness, pore size distribution, and other phenotypic parameters.

### RNA-seq analysis

Total RNA was isolated from the frozen pericarp of two sweet corn samples with three replications using the FastQuant RT Kit (Takara, Dalian, China) according to the manufacturer’s instructions. The purity, concentration, and integrity of the extracted RNA samples were evaluated using Nanodrop, Qubit 2.0, and Agilent 2100, respectively. Subsequently, 4 μg of each RNA sample was utilized for library construction employing the Illumina HiSeq X-ten platform. The raw reads were generated and stored in FASTQ format post-sequencing, and clean reads were obtained through data quality control and filtering as DEGs were identified using the DESeq2 package with log2 (fold change) ≥ 1 and false discovery rate (FDR) < 0.05 as thresholds. For differentially expressed miRNAs, DESeqv1.18.0 software was used with |log_2_ (fold change)| > 1 and FDR < 0.05 as screening criteria (Love et al., 2014). The clean reads were aligned to the maize B73 (Version 4) reference genome (ftp://ftp.ensemblgenomes.org/pub/plants/release-46/fasta/zea_mays/dna/) using HISAT2 (Kim et al., 2019). The gene expression levels were determined using Fragments Per Kilobase of transcript per Million fragments mapped (FPKM) method, and differentially expressed genes (DEGs) were identified using the DESeq R package. Transcripts with log_2_(foldchange) ≥ 1 and a false discovery rate (FDR) < 0.05 were considered as DEGs.

### Metabolome analysis

The pericarp of sweet corn was subjected to freeze-drying using a Scientz-100F freeze-dryer and then pulverized into a fine powder with a grinder (MM 400, Retsch). 0.1 g of each sample was accurately weighed and dissolved in 0.6 mL of 70% methanol extraction solution. After dissolution, the sample was vortexed every 30 minutes for a total of 5 times to improve extraction efficiency and then left overnight in a refrigerator at 4°C. The extraction was performed as previously described metabolites were identified by comparing their retention time, m/z values, and fragmentation patterns with the HMDB database and MetWare in-house library. The mass tolerance was set to ± 5 ppm. The six samples analyzed include 3 biological replicates of M03 and 3 of M08 at 19 DAP (Chen et al., 2013). The sample extracts were analyzed using the UPLC-ESI-MSMS system with the method described by Wang et al. (2020a). The expression pattern of metabolites refers to the relative content (normalized peak area) in M08 compared to M03, with an absolute log_2_^(fold change)^ greater than 1, a p-value less than 0.05, and a variable importance in the projection (VIP) greater than 1 were classified as differentially accumulated metabolites (DAMs).

### Identification of differentially expressed miRNA and prediction of its target genes

Small RNA libraries were constructed using the small RNA Sample Library Prep Kit v2.0 (Illumina, San Diego, CA, United States) following the manufacturer’s protocol. Total RNA (1.5 μg) was used as the starting material, and small RNA (18–30 nt) was enriched by polyacrylamide gel electrophoresis (PAGE). The total RNA extraction kit (FastQuant RT Kit, Takara) is compatible with small RNA sequencing, and the integrity of small RNA fractions was verified using Agilent 2100 with RIN ≥ 7.0. Raw sequencing reads were filtered to remove spliced, repetitive, and low-quality reads, resulting in clean reads with lengths of 18 to 25 nt. Clean reads were then aligned to the Silva, GtRNAdb, Rfam, and Repbase databases using Bowtie software (Langmead and Salzberg, 2012) to filter out rRNA, tRNA, snRNA, and snoRNA. Then unannotated reads were aligned to the maize B73 reference genome for comparison and analysis based on the miRDeep2 software package to identify known miRNA (Friedländer et al., 2012). At the same time, the structure of miRNA is predicted by extending the number of bases to identify new miRNA. DESeqv1.18.0 software (Anders and Huber, 2010) was used for differential expression analysis of miRNA with log_2_(FC) > 1 and FDR < 0.05. psRNATarget (Dai et al., 2018) was utilized for the prediction of miRNA target genes, which were then verified by the complementary pairing of miRNA-mRNA and alignment with NCBI NR, Swiss-Prot, and Pfam databases to obtain annotation information.

### Verification of RNA-sequencing

Total RNA was individually extracted and reverse transcribed to cDNA from each sample using the FastQuant RT Kit (TaKaRa, Dalian, China). The CFX96 Real-Time System (Bio-Rad) was used for qRT-PCR analysis. All reactions were conducted in 20μL volumes containing 1μL cDNA, 0.6 μL of each gene-specific primer, and the SsoFast EvaGreen Supermix Kit (Bio-Rad). The qRT-PCR protocol included an initial denaturation at 94°C for 1 minute, followed by 40 cycles of 95°C for 10 seconds, 55°C for 10 seconds, and 72°C for 15 seconds. The relative gene expression levels were determined using the 2^−△△CT^ method with actin as the reference gene (Livak and Schmittgen, 2001).

## Results

### Analysis of pericarp thickness differences

**Figure 1:**
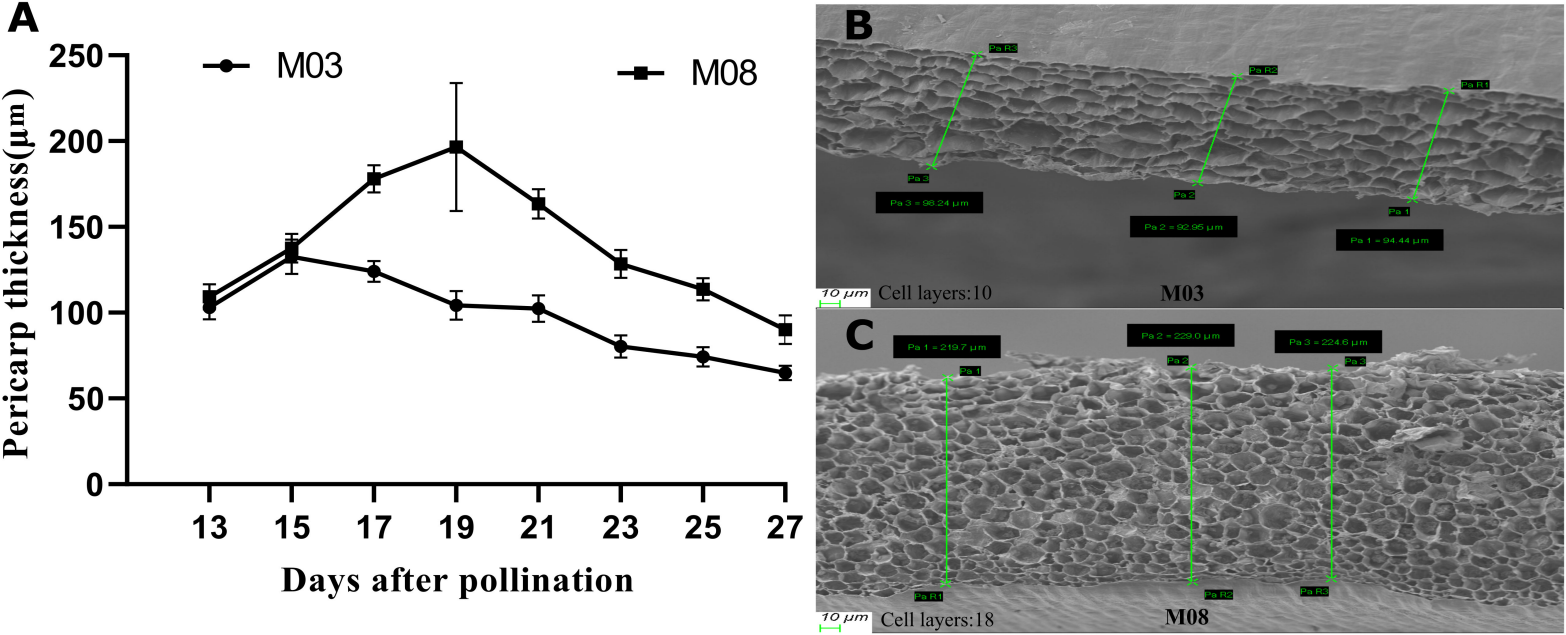
The pericarp thickness differences between M03 and M08 inbred lines at different developmental periods. **(A)** Change process of pericarp thickness of M03 and M08 inbred lines with different pollination days (five replicates in each period). **(B)** SEM of pericarp thickness of M03 at 19 DAP. **(C)** SEM of pericarp thickness of M08 at 19 DAP. The top of the image corresponds to the exocarp, and the bottom corresponds to the endocarp. The more cell layers were presented in **Supplementary Figure S1**.

This study investigated the dynamic changes in pericarp thickness of two sweet corn inbred lines following pollination. Both inbred lines exhibited a pattern of increasing and then decreasing pericarp thickness (**Figure 1A**). At 15 days post-pollination, M03 reached its maximum pericarp thickness of 132.51 ± 10.19 μm, followed by a decline, while M08 reached its peak of 196.53 ± 37.22 μm at 19 days post-pollination, also followed by a decline. After 17 days post-pollination, the pericarp thickness of M08 seeds was significantly greater than that of M03, with the greatest difference observed at 19 days post-pollination. To investigate the mechanism underlying the difference in pericarp thickness, SEM observations of the pericarp thickness of both inbred lines were conducted at 19 days post-pollination (**Figures 1B, C**). The results of the SEM showed that the number of pericarp cell layers of M03 and M08 were 10.6 ± 1.14, and 18.2 ± 1.48, respectively, which indicated that the disparity in pericarp thickness was primarily attributed to variations in the number of pericarp cell layers (**Supplementary Figure S1**).

### RNA sequencing analysis of pericarp thickness between two inbred lines

**Figure 2:**
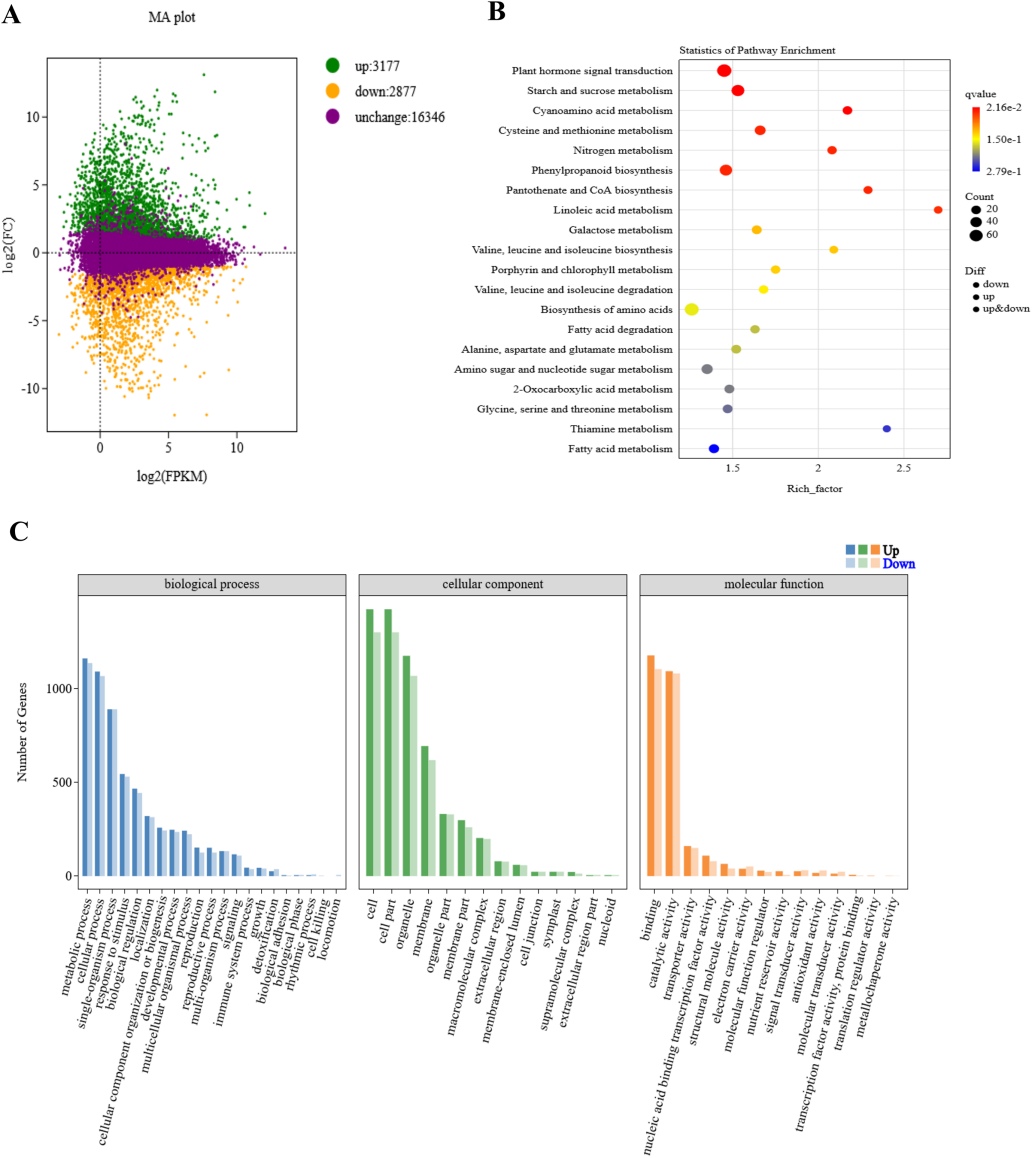
Enrichment analysis of different expressed genes between M03 and M08 lines. **(A)** MA plot displaying DEGs between M03 and M08 lines, with green dots indicating up-regulated genes, orange dots indicating down-regulated genes, and purple dots representing non-significant differences in gene expression levels. **(B)** Enriched KEGG pathways of the target genes, with hotter colors corresponding to lower q-values. The size of the circle reflects the number of DEGs. **(C)** GO annotation of the target genes, with horizontal coordinates indicating different GO terms and vertical coordinates indicating the number of genes.

48.14 Gb of clean reads were obtained from the raw data. The GC content varied from 53.57% to 55.93%, and the Q30 bases accounted for 93.11% to 94.24% of the total bases. The clean reads were aligned to the B73 reference genome of maize, achieving mapping efficiencies ranging from 81.74% to 84.50% for each sample, exceeding the 80% threshold, indicating the accuracy of the transcriptome sequencing data for further analysis (**Supplementary Table S1**). The correlation analysis revealed high intra-group correlation coefficients (r > 0.96) among the biological replicates, demonstrating that the data are of high quality and suitable for further analysis (**Supplementary Figure S2A**). In this study, 6054 DEGs were identified between M03 and M08 lines, with 3177 genes up-regulated and 2877 genes down-regulated (**Figure 2A**). KEGG enrichment analysis showed that DEGs were mainly enriched in pathways related to plant hormone signal transduction, starch and sucrose metabolism, cyanoamino acid metabolism, phenylpropanoid biosynthesis, pantothenate and CoA biosynthesis, and linoleic acid metabolism. This suggests that the differential expression of genes in these pathways, leading to changes in basic metabolites, has a significant impact on the difference in pericarp thickness of sweet corn (**Figure 2B**).

Moreover, Gene Ontology (GO) annotation revealed that DEGs in the biological process (BP) category were primarily associated with metabolic processes, cellular processes, single-organism processes, responses to stimuli, and biological regulation. In the cellular component (CC) category, DEGs were significantly enriched in pathways related to cells, cell parts, organelles, membranes, and organelle parts. In the molecular function (MF) category, DEGs were mainly enriched in binding, catalytic activity, transporter activity, nucleic acid binding transcription factor activity, and structural molecule activity (**Figure 2C**). Five key genes previously identified as QTLs for pericarp thickness were presented in **Supplementary Table S2**, among them, 3 genes were differentially expressed between M03 and M08.

### MiRNA sequencing and key miRNA screening based on the target genes

A total of 76.87 M clean reads were aligned to different databases to classify and annotate small RNAs following miRNA sequencing. The results indicated that the majority of the clean reads were unannotated (66.03%-77.21%), followed by rRNA (16.49%-27.66%), repetitive reads (3.79%-4.92%), tRNA (1.32%-2.29%), and snoRNA (0.09%-0.23%). The unannotated reads were aligned to the B73 maize reference genome, with an alignment efficiency of 51.73%-53.38% for each sample (**Supplementary Table S3**). The high correlation coefficients (r > 0.96) between biological replicates indicate the robustness of the data (**Supplementary Figure S2B**). Known and novel miRNAs were identified using miRDeep2 software, with a total of 248 miRNAs predicted across all samples, including 155 known miRNAs and 93 newly detected miRNAs (**Supplementary Table S4**). The base preference of miRNAs can be determined by analyzing the proportion of miRNA bases. The first base of known miRNAs is predominantly guanine (G), while the first base of newly predicted miRNAs is adenine (A), indicating a significant difference. The 23rd base of known miRNAs shows a strong preference for cytosine (C) and uracil (U), while the 25th base of newly identified miRNAs is exclusively guanine (G). The base preferences at the 5’ end and various positions for known and newly identified miRNAs are illustrated in **Supplementary Figure S2**.

**Figure 3:**
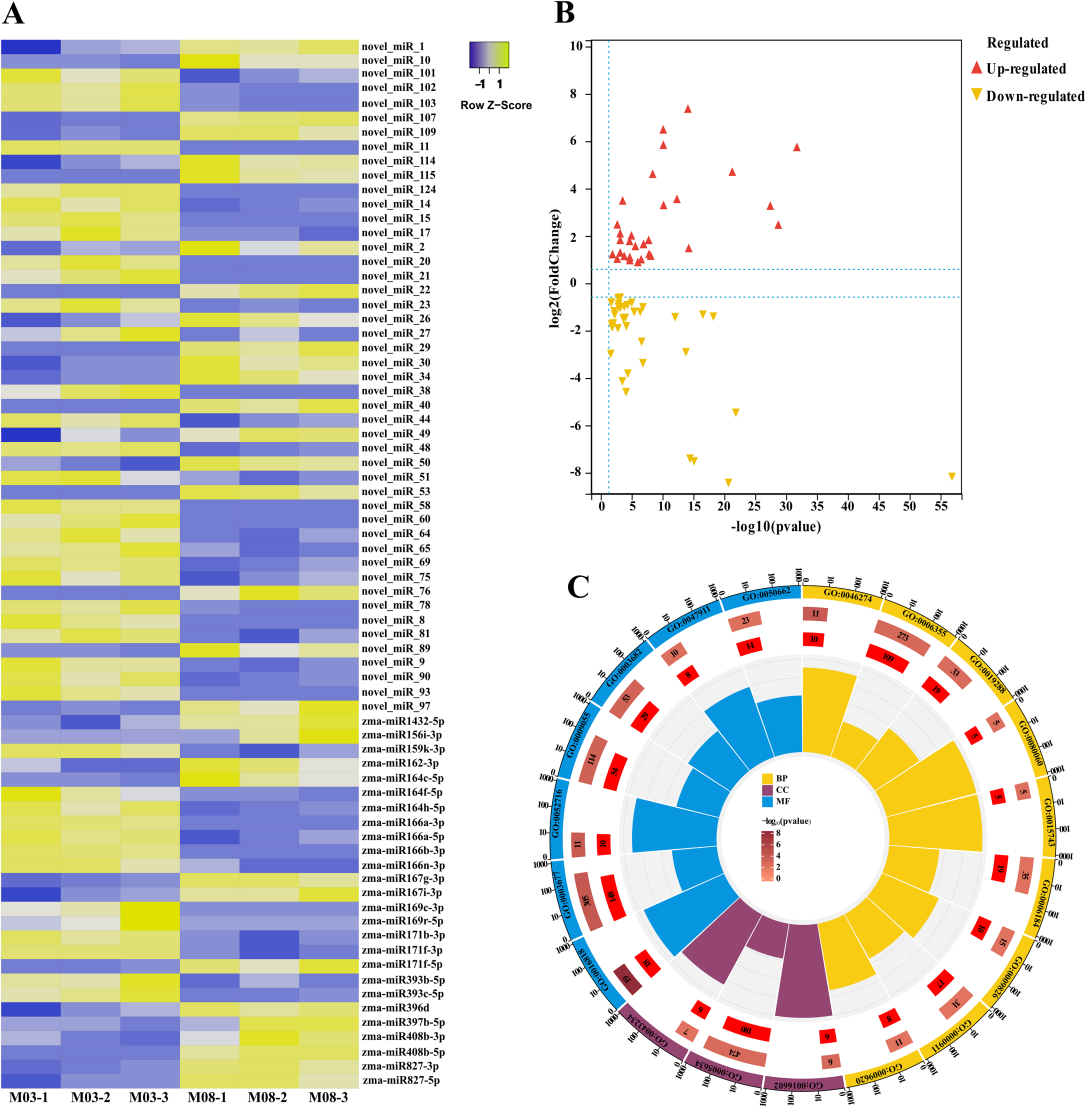
Expression patterns of miRNAs that are differentially expressed between two inbred lines and the GO enrichment analysis of their target genes. **(A)** Expression patterns of 73 miRNAs that are differentially expressed between the M03 and M08 lines. **(B)** Volcano plot illustrating the differential expression of miRNAs between the M03 and M08 lines, where red dots represent up-regulated miRNAs and yellow dots represent down-regulated miRNAs. **(C)** GO enrichment analysis of the target genes of the differentially expressed miRNAs. Different colors are used for different categories, with yellow indicating Biological Process (BP), blue indicating Molecular Function (MF), and purple indicating Cellular Component (CC). Moving from the outer to the inner circles, the first circle represents the GO id, the length of the bar in the second circle corresponds to the number of background genes, the third circle corresponds to the number of target genes, and the polar bar chart in the fourth circle represents the rich factor.

To compare the differentially expressed miRNAs between M03 and M08 on the same pollination day, a threshold of |log2 (fold-change)| > 1 and FDR < 0.05 was used to filter the differential miRNAs, resulting in the identification of 73 differentially expressed miRNAs (**Figure 3A**). Among them, 32 miRNA were up-regulated and 41 were down-regulated (**Figure 3B**). Based on the sequence information of miRNAs, the psRNATarget was utilized to anticipate target genes, resulting in 1674 predicted target genes for the 73 differentially expressed miRNAs. The GO enrichment analysis indicated that the target genes were predominantly enriched in the following categories: BP - lignin catabolic process (GO:0046274), regulation of transcription, DNA-templated (GO:0006355), isopentenyl diphosphate biosynthetic process, methylerythritol 4-phosphate pathway (GO:0019288), integument development (GO:0080060), malate transport (GO:0015743), obsolete GTP catabolic process (GO:0006184), unidimensional cell growth (GO:0009826), cytokinesis by cell plate formation (GO:0000911), response to fungus (GO:0009620); CC - CCAAT-binding factor complex (GO:0016602), nucleus (GO:0005634), protein complex (GO:0043234); MF - hydrolase activity, acting on acid anhydrides, in phosphorus-containing anhydrides (GO:0016818), DNA binding (GO:0003677), hydroquinone:oxygen oxidoreductase activity (GO:0052716), electron carrier activity (GO:0009055), chromatin binding (GO:0003682), galacturan 1,4-alpha-galacturonidase activity (GO:0047911), coenzyme binding (GO:0050662) (**Figure 3C**, **Supplementary Table S5**).

**Figure 4:**
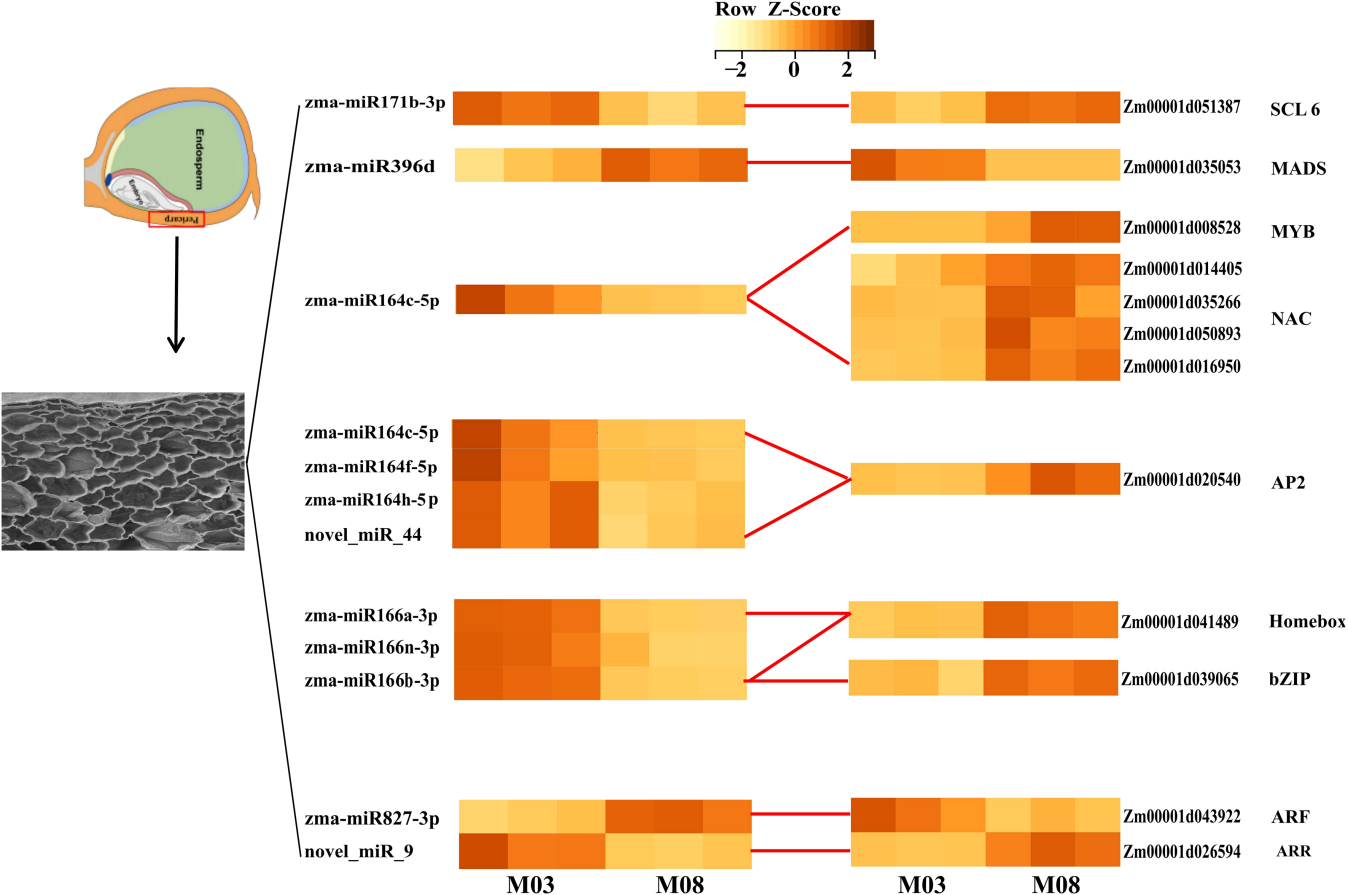
Heatmap of key differentially expressed miRNAs and their target transcription factors. Rows represent miRNAs and TFs, columns represent samples (M03 and M08 with three replicates). The color scale indicates log_2_ (fold change) of expression levels. Darker colors indicate higher expression levels.

miRNAs commonly suppress their targets. In order to identify more robust miRNA-mRNA pairs, correlation analysis of miRNA and target gene expression was performed using RNA-seq and miRNA-seq data from 3 biological replicates of M03 and M08 at 19 DAP. A total of 68 miRNA-mRNA pairs exhibited significantly negative correlations, with an average correlation coefficient of -0.56 (p <0.05, Spearman correlation) (**Supplementary Table S6**). We considered these pairs to be reliable and focused our further analyses on them. Annotation analysis of the target genes identified several key transcription factors. For instance, *zma-miR164c* is down-regulated in M08, regulating the up-regulation of 4 NAC and 1 MYB transcription factors in M08, *zma-miR164c*, *zma-miR164f*, *zma-miR164h*, and *novel_miR_9* negatively regulated an AP2 transcription factor, *zma-miR166a*, *zma-miR166n* and *zma-miR166h* are down-regulated in M08, with its target genes homeobox transcription factor upregulated; *novel_miR_9* and *zma-miR827* each regulate an ARR and ARF, with *novel_miR_9* down-regulated and *zma-miR827* up-regulated in M08, leading to one ARR being up-regulated and the other ARF down-regulated in M08. *zma-miR171b* is down-regulated in M08, and its target gene scarecrow-like 6 (*SCL6*) is up-regulated, indicating that *zma-miR171b* negatively regulates *SCL6* expression (**Figure 4**). These transcription factors are differentially expressed in the pericarp play different roles in the downstream regulatory regions of their target genes, thereby having a significant impact on pericarp thickness.

### Metabolome analysis of pericarp thickness differences

**Figure 5:**
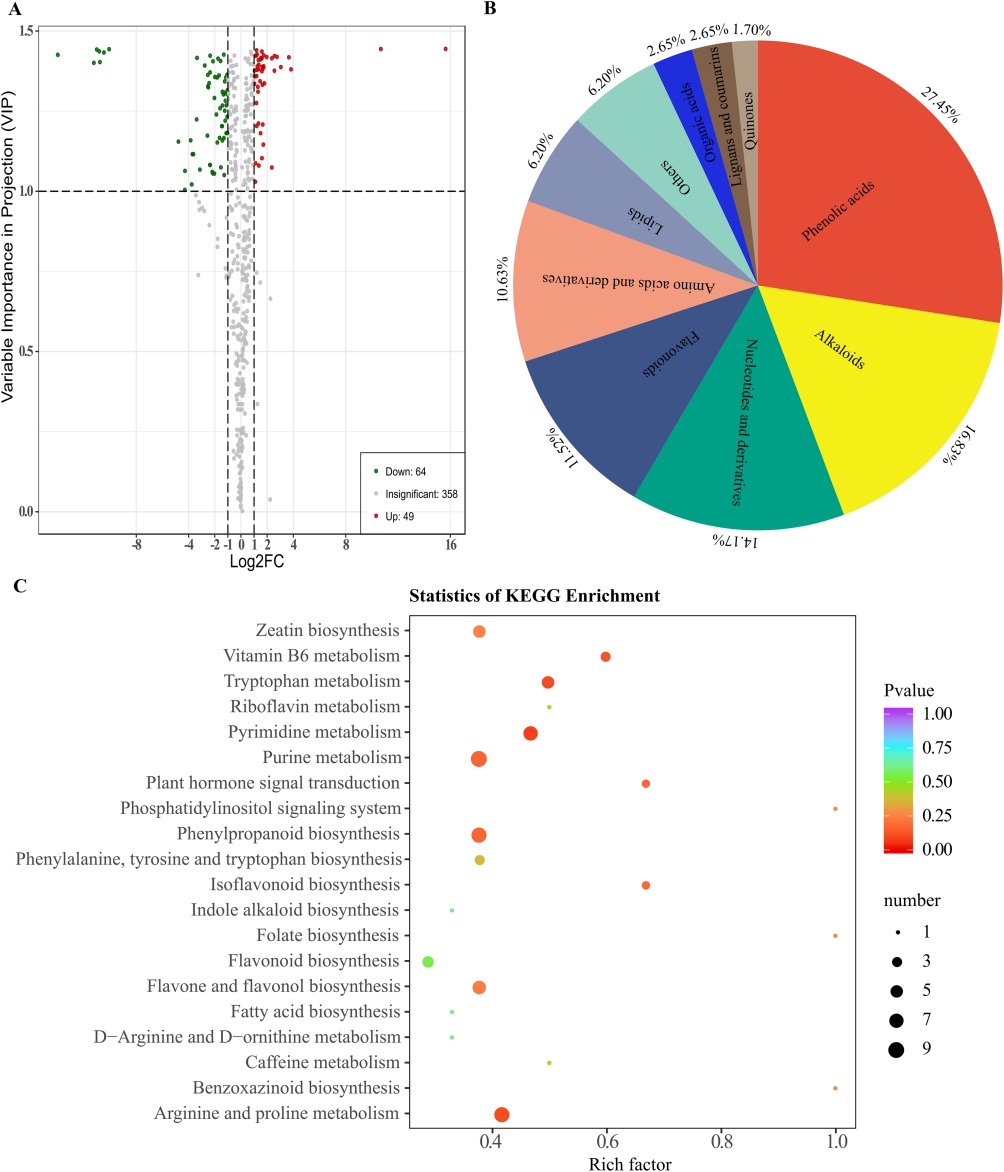
Enrichment analysis of accumulated metabolites between M03 and M08 lines. **(A)** Volcano plot displaying DAMs between M03 and M08 lines, where red dots represent up-regulated DAMs and yellow dots represent down-regulated DAMs. **(B)** Primary classification of the DAMs identified between m03 and M08 groups. **(C)** Enriched KEGG pathways of the DAMs, with hotter colors corresponding to lower p-values. The size of the circle reflects the number of DAMs.

Based on both public metabolic databases (HMDB) and MetWare Company’s in-house metabolic database, the detected metabolites were compared to conduct a qualitative analysis of the metabolites in the samples. A total of 471 metabolites were detected in the six samples. By comparing the metabolites between groups M03 and M08, differential metabolites were selected based on VIP ≥1 and fold change criteria (FC ≥2 or FC ≤ 0.5). In total, 113 DAMs were identified, with 64 metabolites showing down-regulation and 49 metabolites showing up-regulation in M08. (**Figure 5A**, **Supplementary Table S7**). Among these DAMs, the phenolic acids group had the highest number at 31, followed by alkaloids with 19, nucleotides and derivatives with 16, flavonoids with 13, amino acids and derivatives with 12, lipids with 7, others with 7, organic acids with 3, lignans and coumarins with 3, and quinones with the least at 2 (**Figure 5B**). KEGG pathway enrichment analysis was conducted to clarify the biological functions of these DAMs, the results showed that 113 DAMs were predominantly enriched in pathways including zeatin biosynthesis, plant hormone signal transduction, vitamin B6 metabolism, phenylpropanoid biosynthesis, tryptophan metabolism, isoflavonoid biosynthesis, and flavone and flavonol biosynthesis (**Figure 5C**).

### Joint transcriptome and metabolome analysis of pericarp thickness

**Figure 6:**
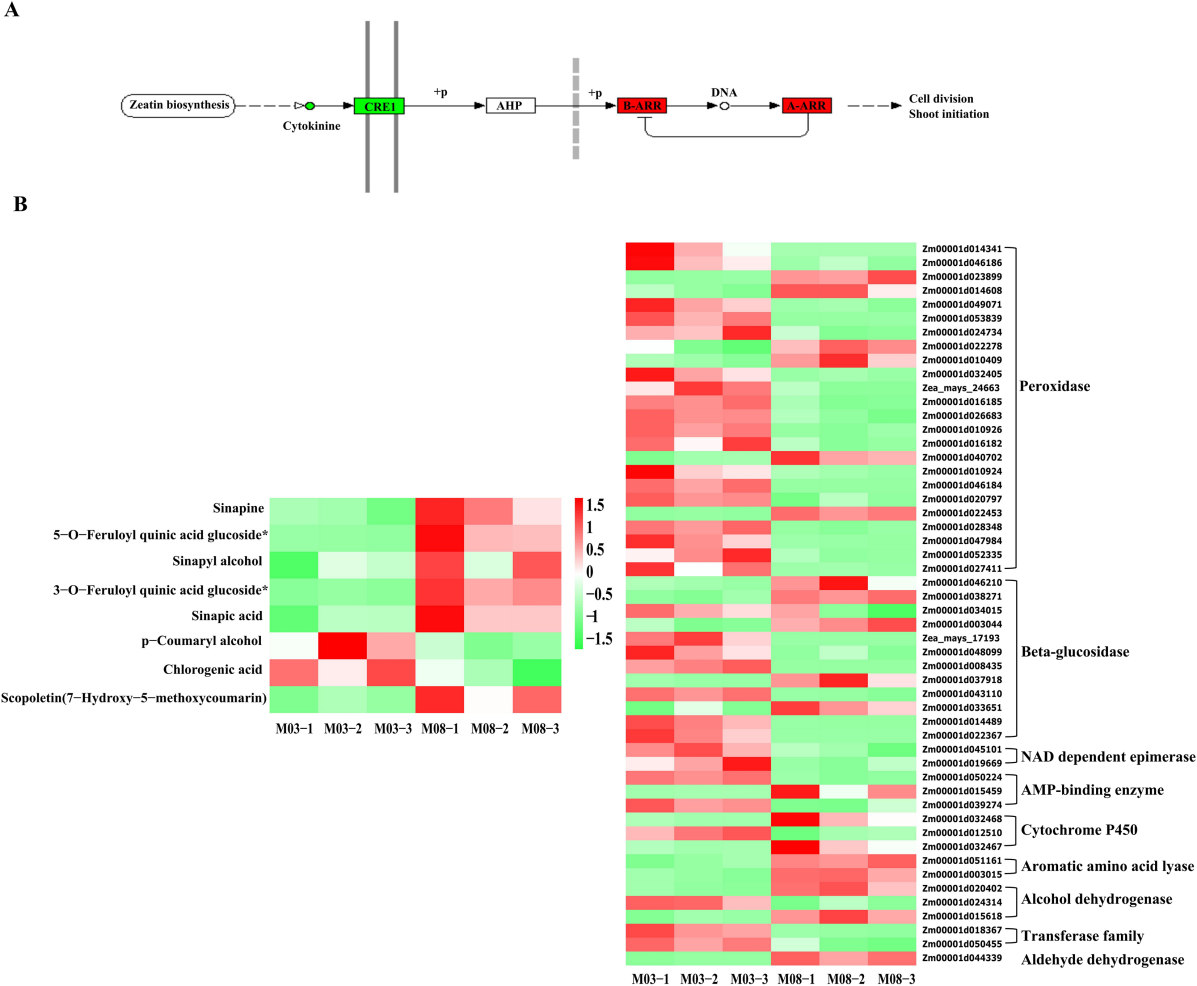
Combined transcription-metabolism analysis reveals key genes and metabolic pathways. **(A)** cytokinin pathway, red is up-regulated, green is down regulated, circle indicates metabolic substances, rectangle indicates genes. **(B)** Key metabolites and key gene expression profiles of phenylpropanoid biosynthesis. M03-1/2/3 and M08-1/2/3 represent three biological replicates of M03 and M08 at 19 DAP.

The joint analysis of DEGs and DAMs associated with the thickness difference of sweet corn pericarp revealed that two common metabolic pathways were significantly enriched in the KEGG functional enrichment. The common enrichment of the plant hormone signal transduction pathway in both transcriptome and metabolome suggests its significant role in in regulating the genetic development of corn pericarp thickness. Subsequent correlation analysis focused on this pathway, identifying the involvement of trans-zeatin and trans-zeatin O-glucoside in plant hormone signal transduction. The analysis of the expression of various metabolites indicated that both trans-zeatin and trans-zeatin O-glucoside were down-regulated in M08. At the same time, the KEGG functional enrichment analysis found that 5 DEGs were involved in the signal transduction of cytokinins. Among them, *Zm00001d019555, Zm00001d025472, Zm00001d026594*, and *Zm00001d045112* were up-regulated, while *Zm00001d042312* was down-regulated in M08. According to the gene annotation data, *Zm00001d019555*, *Zm00001d025472*, and *Zm00001d026594* are classified as A-ARRs, *Zm00001d045112* is a B-ARR, and *Zm00001d042312* is a *HK3* (Histidine kinase 3) that serves as a direct receptor for cytokinins. Cytokinins are down-regulated in M08, and their receptor *HK3* is also down-regulated in M08 expression. As three negative regulators of cytokinins are increased in M08, although one B-ARR is also increased in M08, the overall down-regulation of cytokinins is consistent in expression patterns (**Figure 6A**).

In the KEGG functional enrichment analysis of the metabolome, it was identified that 8 metabolites are associated with the phenylpropanoids biosynthesis pathway. Among them, sinapine, 5-O-feruloylquinic acid glucoside, sinapinic alcohol, 3-O-feruloylquinic acid glucoside, sinapinic acid, and scopoletin (7-7hydroxy-5-methoxycoumarin) were up-regulated in M08, while p-coumaryl alcohol and chlorogenic acid were down-regulated. The transcriptome analysis revealed 52 DEGs involved in the phenylpropanoid biosynthesis pathway. To more clearly see the regulatory changes of genes, a heat map was generated to visualize the related genes and metabolites (**Figure 6B**). Annotation of the 52 genes revealed that 24 were classified as peroxidases, with 18 being down-regulated and 6 up-regulated in M08. There were 12 genes annotated as beta-glucosidases, with 7 of them showing down-regulation in M08, leading to the accumulation and up-regulation of 5-O-feruloylquinic acid glucoside and 3-O-feruloylquinic acid glucoside in M08. Two NAD dependent epimerases and two transferases were down-regulated in M08, while two alcohol dehydrogenases were up-regulated, resulting in the down-regulation of p-coumaryl alcohol. Furthermore, two aromatic amino acid lyases and one aldehyde dehydrogenase were up-regulated in M08. Additionally, there were 3 AMP-binding enzymes, with 2 showing down-regulation and 1 up-regulation in M08, and the expression profiles of DEGs were generally in line with the DAMs.

By conducting an interaction network analysis of these DEGs, target genes of differential miRNAs, and DAMs, it can be observed that genes, proteins, and metabolites involved in various metabolic pathways, including the metabolic pathways, benzoxazinoids biosynthesis, plant hormone signal transduction, phenylpropanoids biosynthesis, purine metabolism, biotin metabolism, biosynthesis of secondary metabolites, styrylpyrones, diarylheptanoids and gingerol biosynthesis, alanine, aspartate, and glutamate metabolism, and pyrimidine metabolism, interact with each other.

In order to confirm the accuracy of the transcriptome sequencing results in this experiment, 9 genes were chosen at random for validation using RT-qPCR. The findings revealed that the expression patterns of the 9 randomly selected genes were in agreement with the expression profiles obtained from the transcriptome sequencing (**Supplementary Figure S3**).

## Discussion

**Figure 7:**
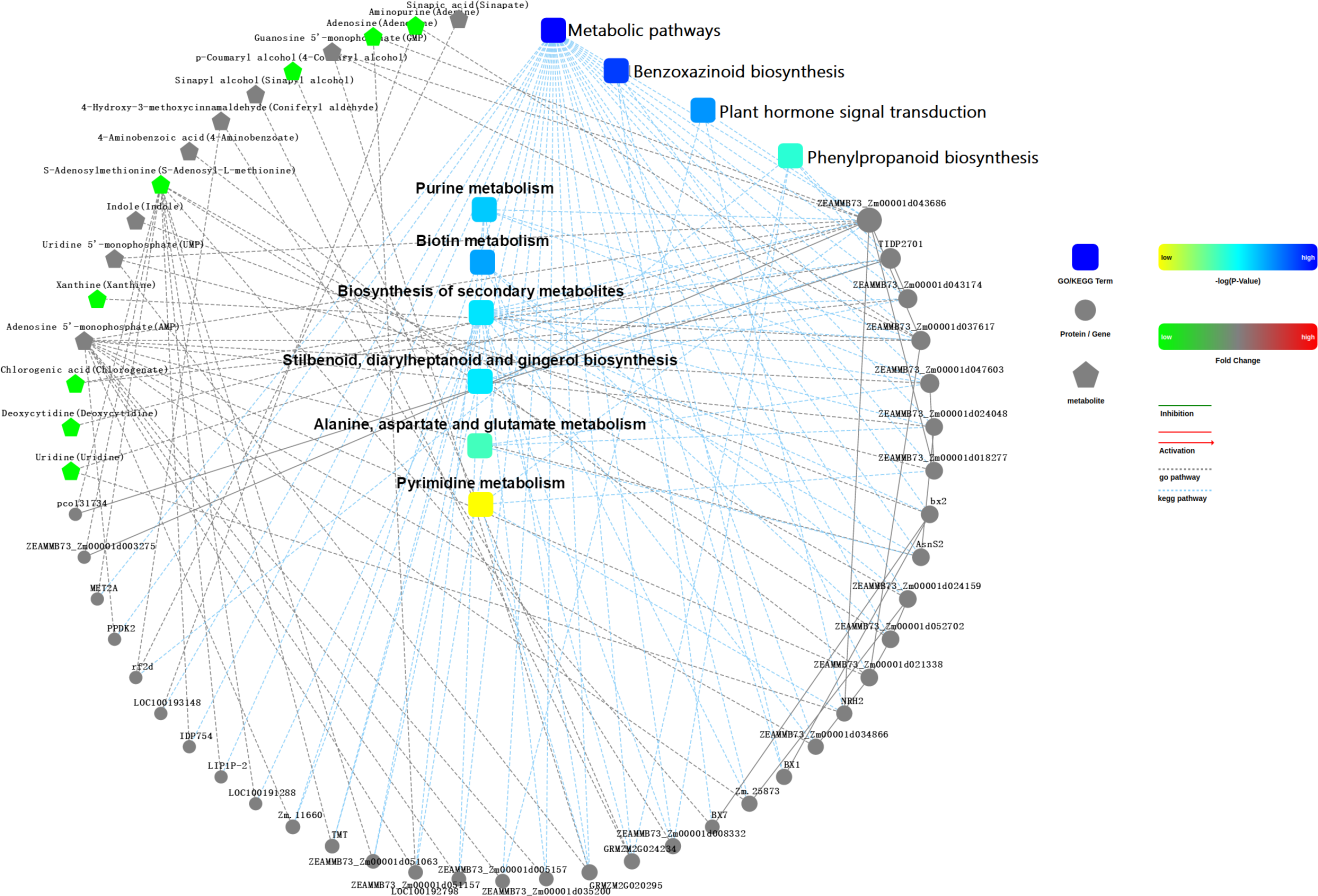
Interaction network analysis of metabolic pathways of genes, proteins and metabolites. A hypothetical interaction network constructed based on KEGG pathway annotations and known interaction databases (omicsbean), linking DEGs, DAMs. ‘Proteins’ in the legend refer to the protein products encoded by the identified DEGs, as no proteomic data were generated in this study. The network highlights that hormone signal transduction and phenylpropanoid biosynthesis are core interconnected pathways.

A major factor in determining the flavor quality of sweet corn is the thickness of the pericarp. Due to the complexity of the pericarp thickness trait, despite the identification of various potential genes in previous studies, no conclusive genes have been successfully cloned thus far, leading to limited understanding of the genetic control involved in this trait (Wu et al., 2020; Gong et al., 2024a). Our study represents the first comprehensive integration of phenotypic, transcriptomic, small RNA, and metabolomic analyses to elucidate the molecular mechanisms underlying sweet corn pericarp thickness. The key findings collectively reveal a sophisticated, multi-layered regulatory network that coordinates cellular, hormonal, and metabolic processes to determine this crucial quality trait (**Figure 7**). The key findings from this study, including the identification of differentially expressed genes (DEGs), metabolites (DAMs), and miRNAs, along with their interactions, offer novel insights into the genetic regulatory network governing this trait.

The SEM analysis revealed that the difference in pericarp thickness between the thin-pericarp line (M03) and the thick-pericarp line (M08) is primarily due to variations in the number of cell layers (**Supplementary Figure S1**). This phenotypic observation aligns with the transcriptomic and metabolomic data, which identified 6,054 DEGs and 113 DAMs enriched in key pathways such as plant hormone signal transduction and phenylpropanoid biosynthesis (**Figure 2B**, **Figure 5C**). The study focuses on two inbred lines with extreme pericarp thickness phenotypes, which helps to identify core regulatory factors but may limit the generalizability of results. These two lines are not near-isogenic lines but share high genetic similarity (82.3%), minimizing the interference of unrelated genetic differences. Future studies should validate the key findings in more germplasm resources. Additionally, the observed molecular differences are correlative, and functional validation is needed to confirm causality.

Plant hormone signal transduction has a direct impact on plant growth and developmental processes. This study identified 118 genes involved in plant hormone signal transduction, with 39 genes participating in auxin signal transduction, including 22 AUX/IAAs, 10 ARFs, and 7 GH3s. ARFs and AUX/IAAs are two different kinds of transcription factors that control auxin signal transduction (Weijers and Friml, 2009; Hayashi, 2012). Auxin levels have a significant effect on cell growth, low concentrations of auxin can promote cell elongation while high concentrations can stimulate cell division and inhibit elongation (Jones et al., 1998; Chen, 2001). The majority of AUX/IAA genes were down-regulated in M08, along with 6 up-regulated ARFs, 4 down-regulated ARFs, and 7 down-regulated GH3s in M08, suggesting a potential association between auxin signal transduction-related genes and pericarp thickness differences. Cytokinins (CTK) promoting cell division and differentiation by regulating the cell cycle at the G1S and G2M phases (Schaller et al., 2014). A-ARRs and B-ARRs are the two categories of CTK response regulators (ARRs), B-ARRs act as positive regulators of CTK, while A-ARRs function as negative regulators that can inhibit the activity of B-ARRs (Rashotte et al., 2006). In this study, 3 A-ARRs and 1 B-ARRs were identified up-regulated in M08. Metabolomic analysis revealed down-regulation of CTKs in M08, as well as down-regulation of their receptor *HK3*, along with 3 up-regulated negative regulators of CTKs, consistent with the transcriptome analysis, indicating that genes involved in CTK signal transduction may regulate changes in pericarp thickness. The down-regulation of CTKs and their receptor HK3 in M08 (thick pericarp line) seems contradictory to the traditional role of CTKs in promoting cell division. However, we propose that this may be due to the tissue-specific and temporal regulation of CTK signaling in pericarp development. In the traditional understanding, the cell division-promoting effect of CTKs refers to accelerating the division rate. However, in certain tissues, the core function of CTKs is to initiate the cell division termination program and halt cell division. When CTK concentrations decrease, the division termination program is delayed, leading to an extended cell division cycle (rather than an accelerated division rate) and a subsequent increase in the total number of cells. In sweet corn pericarp, CTK signaling may primarily regulate cell division termination: reduced CTK levels could delay the cessation of cell proliferation, leading to more cell layers. Additionally, the up-regulation of 3 A-ARRs (negative regulators of CTK signaling) in M08 may further enhance this effect by inhibiting the feedback inhibition of CTK signaling, thereby maintaining prolonged cell division. This hypothesis is supported by previous studies showing that moderate inhibition of CTK signaling can extend the cell division period in plant tissues (Ioio et al., 2008; Sreenivasulu et al., 2008; Pielot et al., 2015). Gibberellins (GA) primarily control cellular growth by influencing the stability of *DELLA* proteins (Band et al., 2012). This research has identified 5 genes associated with the GA signal transduction pathway, including 2 encoding DELLA proteins, 1 being up-regulated and 1 down-regulated. This suggests that further analysis is needed to understand how these genes contribute to the development of pericarp thickness. This study identified 22 genes associated with the ABA signal transduction pathway, of which 17 genes were up-regulated in M08, suggesting their potential role in regulating pericarp thickness. ABA can regulate stomatal closure, which may impact pericarp thickness. The plasma membrane receptors *BRI1* and *BAK1* detect the signaling of brassinosteroids (BR). Numerous positive (*BSK1*, *BSU1, PP2A, and CDG1*) and negative regulatory factors (*BKI1, BIN2, MSBP1*) influence the activity of the *BZR1* and *BES1* family transcription factors, which affect the expression of hundreds to thousands of distinct BR downstream genes (Wang et al., 2008; Kim et al., 2009). In this research, eight genes were found to be part of the brassinosteroid signaling transduction pathway, with 4 showing increased activity and 4 showing decreased activity in M08. The thickness of the pericarp is greatly influenced by the quantity of cells, and BR primarily affects cell division and elongation, indicating that these genes might be involved in controlling the development of the pericarp. The identification of these hormone signal transduction genes advances our knowledge of how hormones control the growth of the pericarp of sweet corn. Our findings are consistent with Xiong et al. (2022), who reported that hormone signaling pathways are involved in sweet corn pericarp thickness regulation. Compared to single-omics studies, our integrated approach reveals the coordinated regulation of genes, miRNAs, and metabolites, providing a more comprehensive regulatory network.

**Figure 8:**
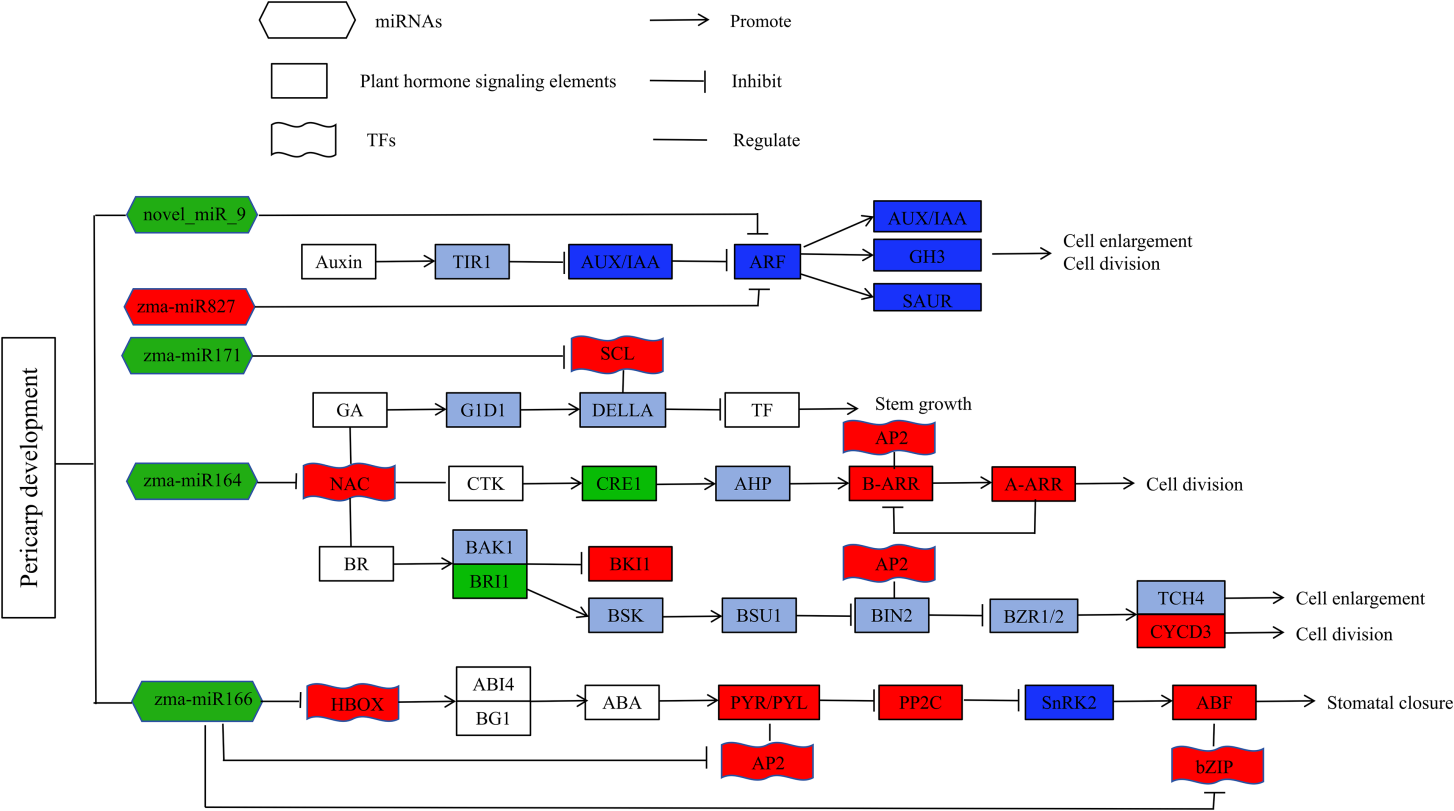
Hypothesis genetic network for plant hormones regulate pericarp thickness in sweet corn. Red color indicated that the related genes were up-regulated, green color means that the related genes were down-regulated, blue color means that the related genes were both up- and down-regulated, and purple indicated that the up or down-regulation relationship of the related gene has not been determined.

Key miRNAs were found to target and regulate different transcription factors. For example, zma-miR164c was down-regulated in M08, leading to the up-regulation of 4 NAC and 1 MYB transcription factors. There have also been reports of miR164 controlling NAC transcription factors in other plants (Fang et al., 2014; Wang et al., 2020b). Most of the reported NAC transcription factors are associated with plant responses to abiotic stresses (An et al., 2018; Hao et al., 2020), and some researchers believe that NAC transcription factors can regulate GA/BR and CTK signal transduction, thereby affecting cell elongation and division (Kim et al., 2006; Shahnejat et al., 2016). *Zma-miR166a* was down-regulated in M08, with the corresponding Homeobox-leucine zipper protein (*HD-ZIP*) up-regulated. Studies have found that in Arabidopsis, *STTM165/166* promotes the degradation of *miRNA165/166*, leading to increased expression of their target gene *HD-ZIP* (Jia et al., 2015). Other researchers have found that overexpression of *STTM165/166* lead to decreased expression of *miR165/166d*, and the up-regulation of *miR165/166* target genes directly promotes the accumulation of *ABI4* and *BG1*, thereby regulating ABA (Yan et al., 2016). Additionally, *zma-miR167g* and *zma-miR827* each regulated an ARF, with *zma-miR167* down-regulated and *zma-miR827* up-regulated in M08. ARF is an auxin response factor that has the ability to increase auxin downstream gene expression (Weijers and Friml, 2009). Moreover, *zma-miR171b* was down-regulated in M08 and negatively regulated the up-regulation of *scarecrow-like 6* (*SCL6*) gene expression. *SCL* belongs to the *GRAS* gene family, and in Arabidopsis, the miR171 target gene *SCL* regulates GA expression in two ways: one is by binding to GT cis-elements to regulate GA expression (Ma et al., 2014), and the other is by antagonizing the GA signal repressor DELLA to promote GA expression (Zhang et al., 2011). This study also identified novel miRNAs that might regulate the thickness of the sweet corn pericarp via various mechanisms. Based on this, we propose a genetic network hypothesis for how plant hormones regulate pericarp thickness in sweet corn (**Figure 8**).

Previous research has shown that maize susceptibility to pests and diseases is correlated with the level of phenylpropanoid compounds in the kernel (Santiago and Malvar, 2010). High concentrations of phenylpropanoid compounds significantly reduced disease levels (Sampietro et al., 2013). A study explored the association between phenylpropanoid content and pericarp thickness using a mutant of the *P1* (pericarp color 1) gene, suggesting that the *P1* gene increases pericarp thickness by regulating the accumulation of phenylpropanoid compounds, which in turn enhances disease resistance (Landoni et al., 2020). In our study, it was discovered that eight metabolites participate in the phenylpropanoid biosynthesis pathway, including sinapine, 5-O-feruloylquinic acid glucoside, 3-O-feruloylquinic acid glucoside, scopoletin, sinapinic alcohol, and sinapinic acid, which were found to be up-regulated in M08. In contrast, p-coumaryl alcohol and chlorogenic acid were down-regulated in M08. This suggests that the phenylpropanoid content in M08 is higher, and the pericarp of M08 is significantly thicker than that of M03, indicating that phenylpropanoids can thicken the pericarp of sweet corn, in line with previous research.

However, this study still has some limitations: 1) Only two inbred lines were used, and results need validation in more germplasm; 2) Functional validation of key nodes (e.g., zma-miR164c, ARF, cytokinin receptors) are lacking. Future studies should perform gene editing or overexpression experiments to confirm the role of these candidates in pericarp thickness regulation. Additionally, exploring the temporal dynamics of the regulatory network across different developmental stages will enhance our understanding of pericarp development.

## Conclusion

The phenotypic data observation indicated that the disparity in pericarp thickness was primarily attributed to variations in the number of pericarp cell layers. Our analysis of two inbred lines with notably varying pericarp thicknesses utilized transcriptomics, miRNA, and metabolomics to uncover the mechanisms of pericarp thickness formation. Through the miRNA-mRNA interaction pairs analysis, we identified several key miRNAs, including zma-miR164, zma-miR166, zma-miR827, and zma-miR171b, which regulate the signal transduction of different plant hormones by controlling the expression of their target genes, thus influencing the pericarp thickness in sweet corn. Pericarp growth and development are significantly regulated by genes and metabolites involved in the phenylpropanoid biosynthesis pathway and plant hormone signal transduction, according to integrated transcriptomic and metabolomic research. In the thick-skinned M08, the content of cytokinins was significantly reduced, and there was a significant accumulation of phenylpropanoid compounds such as sinapine, 5-O-feruloylquinic acid glucoside, sinapinic alcohol, 3-O-feruloylquinic acid glucoside, sinapinic acid, and scopoletin (7-hydroxy-5-methoxycoumarin). This study offers fresh perspectives on the processes that underlie the development of sweet corn pericarp thickness.

## Data Availability

The raw sequencing data of transcriptome and miRNA-seq have been deposited in the NCBI Sequence Read Archive (SRA) database under the accession number PRJNA807465 and PRJNA807472.

## Supplementary material

Supplementary Figure 1 Pericarp thickness and cell layer number of M03 and M08 under SEM. **(A–C)** indicated the pericarp thickness and cell layer number of M03. **(D–F)** Pericarp thickness and cell layer number of M08.

Supplementary Figure 2 Correlation analysis of biological replicates for the RNA-seq and miRNA-seq datasets. **(A)** Correlation analysis of the transcriptome data. **(B)** Correlation analysis of the miRNA sequencing data.

Supplementary Figure 3 Distribution of length and base biases of endogenous for known and novel miRNA.

Supplementary Figure 4 Verification of relative expression levels of DEGs was conducted using qRT-PCR.
