# Supplementary figures and images for: Combined transcriptome, metabolome, and miRNA analysis reveals the genetic regulatory network of sweet corn pericarp thickness
Source: Front Plant Sci. 2025 Dec 12;16:1698281. doi: 10.3389/fpls.2025.1698281 (PMC12741122; doi:10.3389/fpls.2025.1698281)

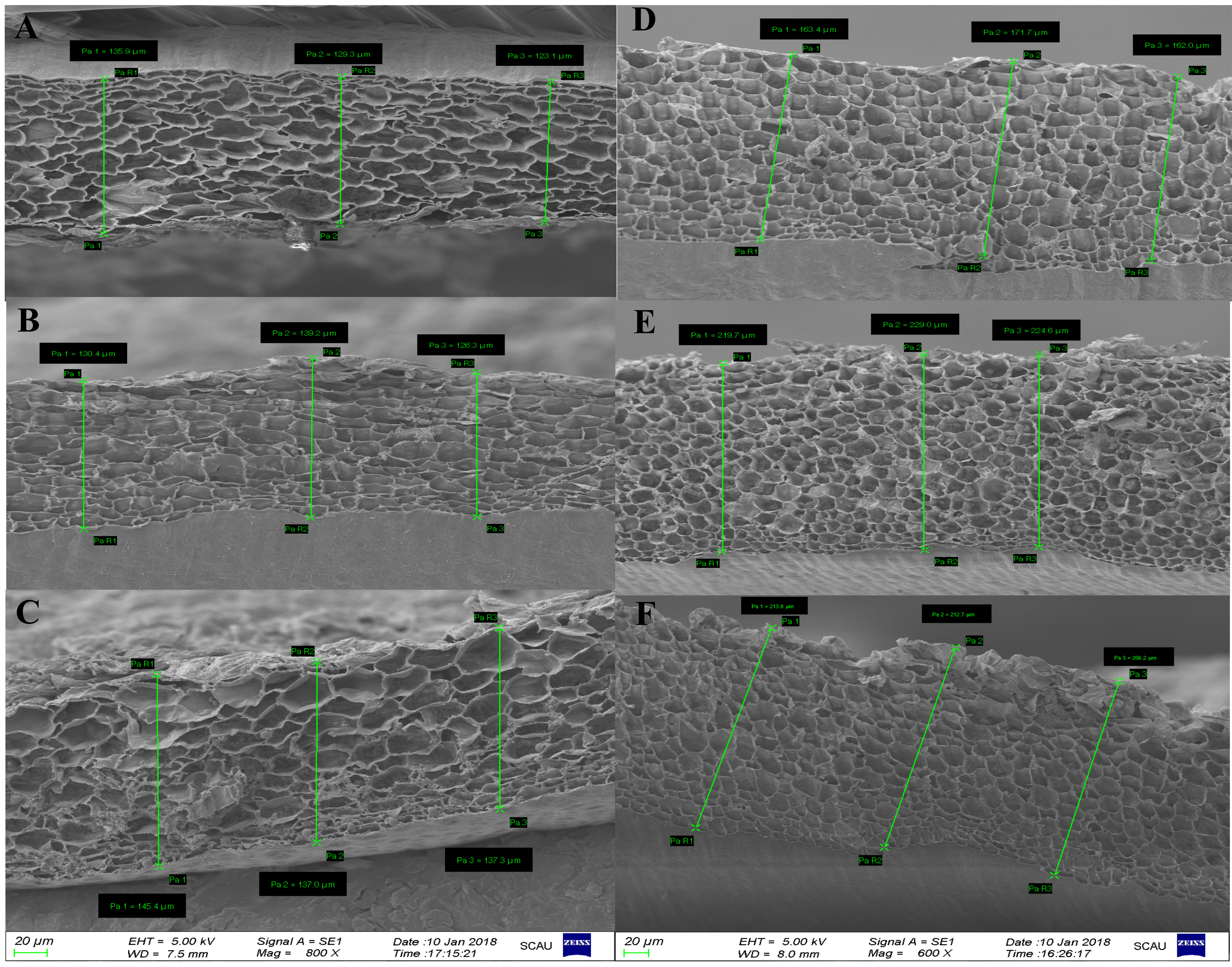

Supplement: Supplementary Figure 1 — Pericarp thickness and cell layer number of M03 and M08 under SEM. (A–C) indicated the pericarp thickness and cell layer number of M03. (D–F) Pericarp thickness and cell layer number of M08. [file Image1.tif]

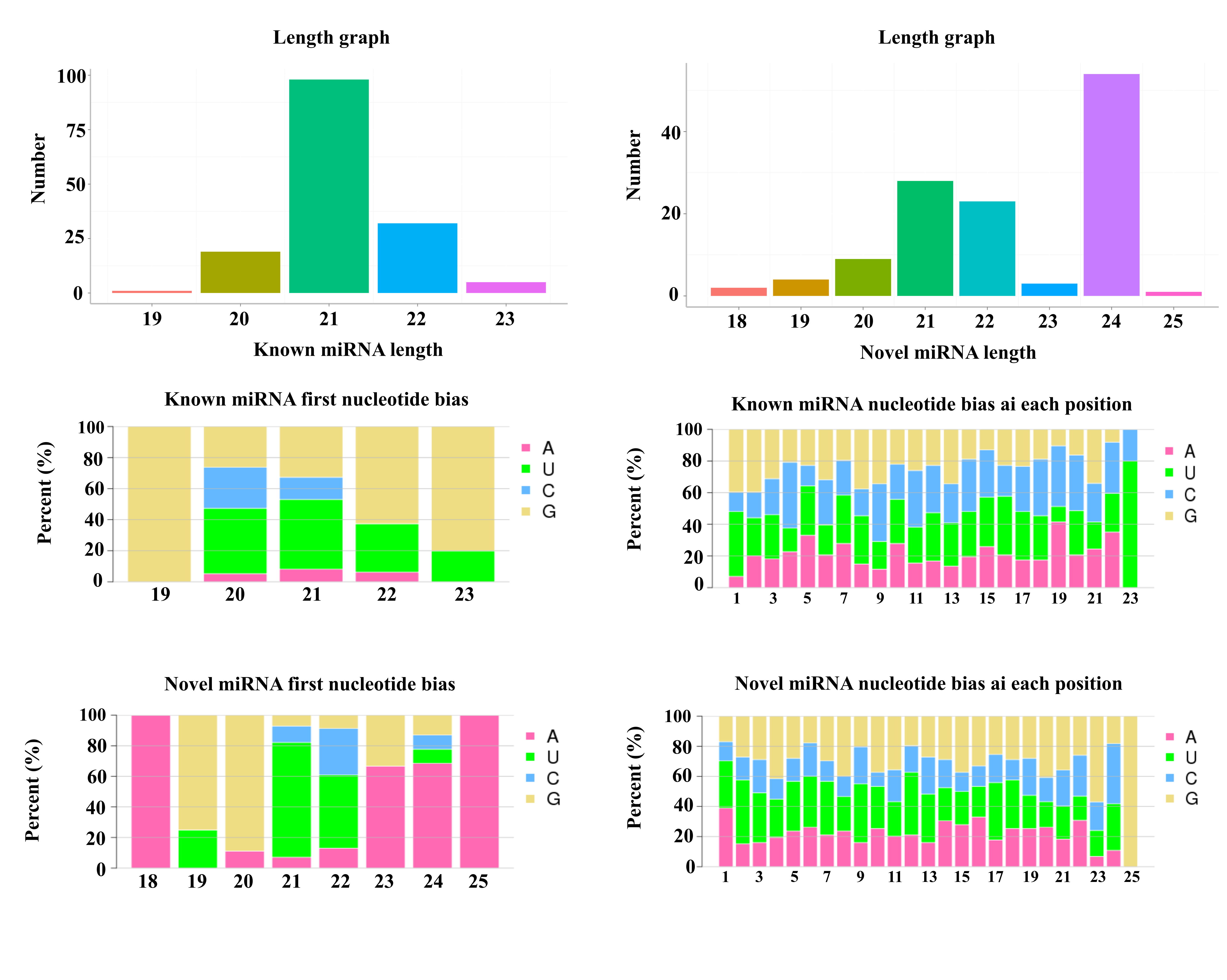

Supplement: Supplementary Figure 3 — Distribution of length and base biases of endogenous for known and novel miRNA. [file Image3.tif]

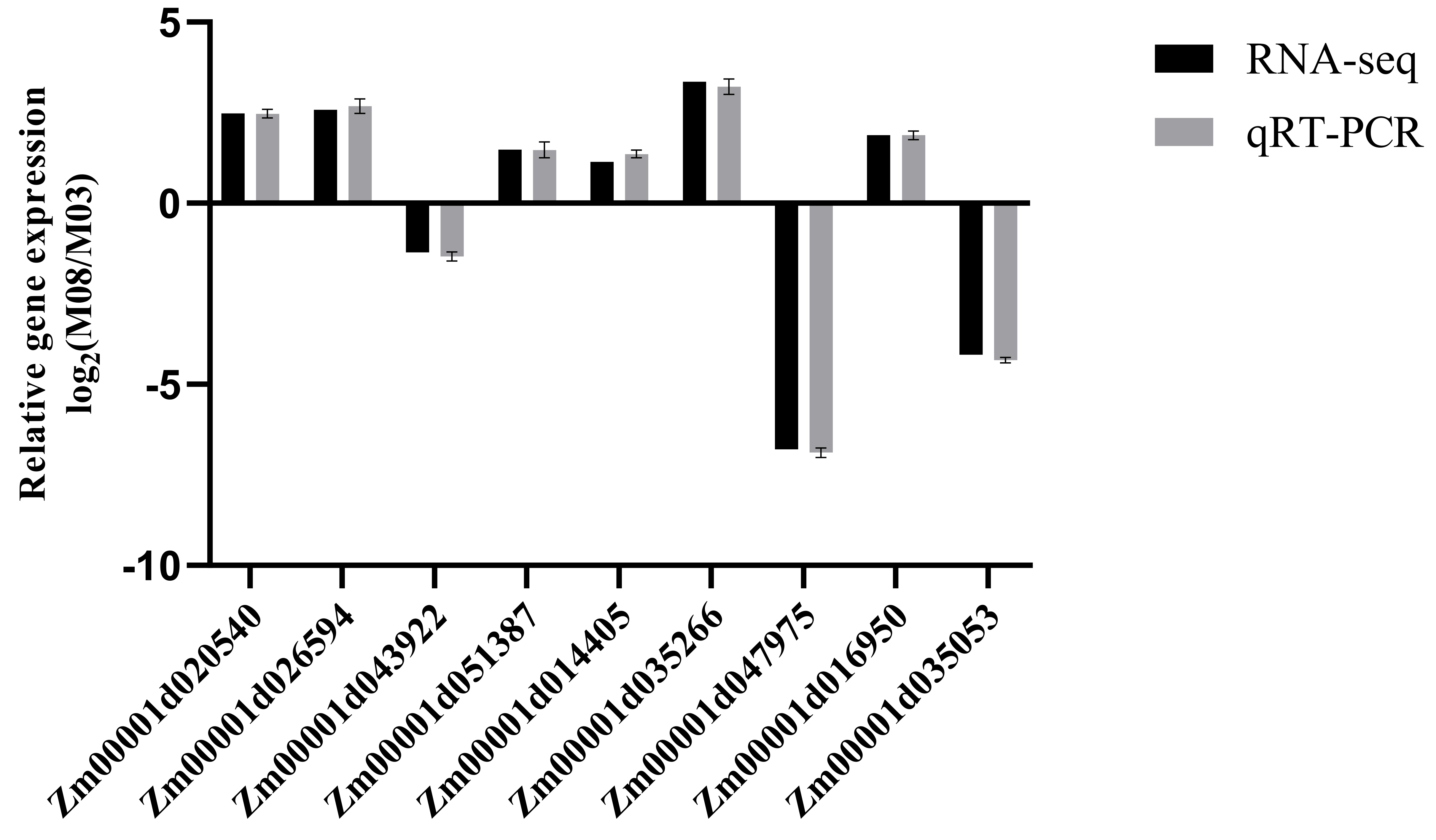

Supplement: Supplementary Figure 4 — Verification of relative expression levels of DEGs was conducted using qRT-PCR. [file Image4.tif]
